# Pituitary neuroendocrine tumors treated with stereotactic radiosurgery

**DOI:** 10.1007/s11060-024-04864-3

**Published:** 2024-10-28

**Authors:** Inhwa Kim, Michael Yan, Michel Sourour, Robert Heaton, Colin Faulkner, Aristotelis Kalyvas, Dana M. Keilty, Michael D. Cusimano, David Payne, Normand Laperriere, David B. Shultz, Saira B. Alli, Gelareh Zadeh, Derek S. Tsang

**Affiliations:** 1 Department of Radiation Oncology, University of Toronto, Toronto, ON Canada; 2 Radiation Medicine Program, Princess Margaret Cancer Centre, University Health Network, Toronto, ON Canada; 3 Division of Neurosurgery, Toronto Western Hospital, University Health Network, Toronto, ON Canada; 4 Division of Neurosurgery, St. Michael’s Hospital, Unity Health Toronto, Toronto, ON Canada

**Keywords:** Radiosurgery for pitNET

## Abstract

**Purpose:**

Pituitary neuroendocrine tumors (pitNETs) are benign tumors that may recur after surgical resection or persist following medical management. The objective of this study was to evaluate outcomes and toxicities of patients with pitNETs treated with stereotactic radiosurgery (SRS) at a single institution.

**Methods:**

We completed a retrospective, single-institution study of patients with pitNETs treated with frame-based, single-fraction, cobalt-60 SRS between September 2005 and June 2023. The primary endpoint was local tumor control. Secondary endpoints included endocrine control (for functional tumors), overall survival, and toxicities.

**Results:**

A total of 88 lesions in 83 patients were treated with SRS. Most lesions (70%) were non-functional tumors. Of the 26 functioning tumors, 6 patients achieved endocrine remission with SRS alone (23%), and the remainder achieved remission with combined medical management. With a median patient follow-up of 4.7 years, no local tumor recurrences were observed with an estimated local control probability of 100%. Two- and five-year overall survival estimates were 97% (95% confidence interval [CI] 89–99) and 95% (95% CI 84–98), respectively. Causes of death were unrelated to PitNET or SRS. Twelve patients (14%) developed hypopituitarism after SRS. Despite the 34 lesions that were ≤ 3 mm from optic structures, no patients developed any optic neuropathy or visual decline post SRS.

**Conclusions:**

SRS is a highly effective modality for recurrent or residual pitNETs. This study observed a local control of 100% with no cases of optic toxicities after a median follow-up of 4.7 years. These observed findings suggest that dose de-escalation may be possible for future treatment of pitNETs.

## Introduction

Pituitary neuroendocrine tumors (pitNET; formerly known as pituitary adenomas) are benign tumors within the sellar region that represent approximately 10% of primary intracranial tumors [1]. PitNETs can present with clinical symptoms of excess hormone secretion (functioning tumors) or mass effect (nonfunctioning tumors). The first-line management for the majority of pitNETs is transsphenoidal surgery with the exception of prolactinomas, which typically undergo first-line medical management with pharmacotherapy to suppress prolactin secretion. Repeat surgical resection, with or without radiation therapy, is typically reserved for persistent or recurrent disease [2].

The primary goal of stereotactic radiosurgery (SRS) is local tumor control, as well as control of hormone secretion in the case of functioning pitNETs. In non-functioning pitNETs, SRS is reported to achieve local tumor control probabilities between 76 and 95% [3]. When considering the endpoint of remission in functioning pitNETs, the success rates are lower, estimated to be approximately 46% with a range of 25–65% depending on type of hormone secretion [4]. Prescription doses range from 12 to 20 Gy for non-functioning tumors, while functioning tumors require higher doses of up to 25 Gy [4].

A consideration when selecting patients for SRS is the proximity of tumor to the anterior visual pathway due to risk of radiation-induced optic neuropathy. It is desirable to have at least 2 mm between tumor and optic structures [5]. In this setting, the accepted maximum tolerance dose to optic structures for a single fraction is typically 10 Gy point dose for a < 1% risk of optic neuropathy [6]. The incidence of optic neuropathy for pitNETs treated with SRS, both non-functioning and functioning tumors, is reported to range from 0 to 7% [4].

Our centre has nearly 20 years of experience with multidisciplinary management of recurrent and residual pitNETs with radiosurgery, where proximity to the optic apparatus (optic nerves, chiasm) is not an absolute contraindication to SRS. The objective of this study was to retrospectively assess the outcomes and toxicities of patients with pitNET treated with radiosurgery.

## Methods

### Study design

We completed a retrospective, single-institution study of patients with pitNETs treated with frame-based, single-fraction, cobalt-60 stereotactic radiosurgery between September 2005 (inception of our gamma knife program) and June 2023 at the Toronto Western Hospital within the University Health Network. Patients were identified from a clinical treatment log. Data was collected from patient charts by 3 independent reviewers (IK, MS, CF) and reviewed by an additional reviewer in cases of uncertainty. The primary endpoint was local tumor control, defined as absence of any sustained radiologic growth of the treated lesion in follow-up as determined by neuro-radiologist review and confirmed by neurosurgeon and/or radiation oncologist review. Secondary endpoints included endocrine control (for functional tumors), overall survival, and toxicities. Endocrine control was collected from the impression of endocrinologists, which was generally defined as the combination of the absence of hormone level elevation (> upper limit of normal), clinical symptomatology from hormonal excess, and need to increase or start medication. This study was reviewed by the research ethics board of the University Health Network (CAPCR ID 23-5325).

### Radiosurgery

Patients were treated with Leksell Gamma Knife 4C prior to 2012 or with Leksell Gamma Knife Perfexion after 2012 (Elekta AB, Stockholm, Sweden). Indications for treatment included tumor progression not suitable for re-resection or hormonal persistence despite prior surgical and medical management. In brief, all patients were initially seen in a multidisciplinary clinic by a neurosurgeon and radiation oncologist. Patients for whom radiosurgery was recommended subsequently underwent magnetic resonance imaging (MRI) within 1 week of planned radiosurgery with contrast-enhanced three-dimensional T1 MP RAGE and T2 SPACE sequences (0.8–1 mm axial slice thickness). On the day of treatment, a Leksell stereotactic frame was applied, followed by high-resolution computed tomography (CT) imaging with 1 mm slice thickness. The MR and CT were registered in GammaPlan (Elekta, AB), which was also used to plan treatments and evaluate dosimetry. Targets and organs-at-risk (chiasm, optic nerves) were contoured by a radiation oncologist with consensus agreement of final volumes after neurosurgeon review. Prescription doses were based on tumor functional status and volume as per institutional guidelines with exact dose to be determined by the treating radiation oncologist and neurosurgeon. Secretory tumors < 14 cc in volume were prescribed 20–25 Gy in 1 fraction. Non-secretory tumors < 4 cc, 4–14 cc and 14.1–30 cc were prescribed 16–18 Gy, 14–16 Gy or 12–14 Gy, respectively. Treatments were planned by a medical physicist to the 50% isodose line. The planning goal for the optic apparatus was < 8–10 Gy to 1 mm^3^, which is less than the goals stated in QUANTEC to minimize risk of optic neuropathy [7]. In cases where optic apparatus doses exceeded this guideline, dose to the region of tumor closest to optic structures was reduced as per treating-physician discretion to meet constraints.

Patients in follow-up underwent clinical assessment, bloodwork, and MR of the sella at 6 months post-treatment, followed by yearly assessments until year 5 post-treatment, every two years until year 10 post-treatment, and then every 2–3 years beyond. Patients with functioning tumors or hypopituitarism following treatment were also seen by an endocrinologist in follow-up. Those with visual symptoms following treatment were subsequently assessed by ophthalmology for possible optic toxicities.

### Statistical analysis

The primary outcome was local tumor control with treated lesion as the unit of analysis. Local failure was defined as growth of the lesion treated with radiosurgery. Baseline characteristics and toxicities were reported descriptively. Overall survival was evaluated using the Kaplan-Meier method with individual patients as the unit of analysis; patients were censored if lost to follow-up. The index time for all analyses was the date of radiosurgery treatment for pitNET. Analyses were done using SAS version 9.4 (Cary, USA). Toxicities were scored using CTCAE (version 5) [8].

## Results

A total of 88 lesions in 83 patients were treated with radiosurgery. Five patients each had two separate lesions treated, one each in left and right sellar regions: Three patients were treated synchronously on the same day, and two patients had two separate treatments (3 and 8 years between radiosurgery treatments). Two patients had prior radiation treatment prior to SRS treatment within our institution: One was SRS, and the second was 46.8 Gy fractionated 3-field using 3DCRT (conformal radiotherapy at an outside institution). The majority of tumors had surgery prior to SRS (98%) with the exception of 2 patients who did not have surgery because they were incidentally detected. Of the 2 patients who did not have prior surgery, one had SRS due to growth and the other continued to have hormonal persistence despite medical management. The median time from surgery to SRS was 4 years. Baseline patient and tumor characteristics are reported in Table 1. Most lesions (70%) were non-functional pituitary tumors. Table 2 shows details of radiosurgery treatments. The most frequently used marginal prescription doses were 18 Gy (28%), 24 Gy (19%), 20 Gy (18%) and 16 Gy (14%). Tumor coverage with 100% (V100) and 95% (V95) of the prescription dose was 99.5% and 99.9%, respectively. Thirty-three tumors had a V100 of less than 99.5%.

There were a total of 26 functioning tumors in 25 patients, as 1 patient had 2 distinct functioning tumors. Six patients achieved endocrine remission with SRS alone (23%). Four tumors were corticotrophs, and 2 tumors were somatotrophs. The majority required a combination of SRS and medical management to achieve remission (77%). Fifteen tumors were somatotrophs (75%), 2 tumors were corticotrophs (10%), and 3 tumors were prolactinomas (15%).

**Table 1:** Baseline characteristics (*N* = 83 patients, *N* = 88 lesions)

Characteristic	Value (%)
Median age at diagnosis, years (IQR)	44 (15)
Median age at SRS, years (IQR)	56 (21)
Sex	
Male	40 (48%)
Female	43 (52%)
Prior radiation	4 (5%)
Prior medical therapy	25 (28%)
Prior surgery	86 (98%)
Physiology	
Functioning	26 (30%)
Non-functioning	62 (70%)
MIB-1 labelling index, % (IQR)	3 (3.5)
Histology	
Null cell	7(8%)
Corticotroph	11 (13%)
Gonadotroph	24 (29%)
Somatotroph	18 (22%)
Prolactinoma	5 (6%)
Oncocytoma	3 (4%)
Not specified	15 (17%)

IQR: Interquartile range; SRS: stereotactic radiosurgery

**Table 2:** Treatment characteristics of lesions (*N* = 88 lesions)

Characteristic	Median Value (IQR)
Prescribed dose, cGy	1800 (400)
1200	3 (3.41%)
1400	5 (5.68%)
1500	1 (1.14%)
1600	12 (13.64%)
1800	25 (28.41%)
1900	1 (1.14%)
2000	16 (18.18%)
2100	2 (2.27%)
2200	3 (3.41%)
2300	2 (2.27%)
2400	17 (19.32%)
2500	1 (1.14%)
Prescription isodose line, %	50 (4.5)
Calibration dose rate on day of treatment, Gy/min	2.5 (1.1)
Total shots, n	17 (8)
Tumor volume, cm^3^	1.7 (1.8)
Tumor max diameter, mm	20.0 (8.7)
Total beam-on time, min	90.4 (49.2)
Tumor V100, cm^3^	99.5 (1)
Tumor V95, cm^3^	99.9 (0.4)
Closest tumor distance from optic structures, mm	4.0 (3.3)
Max dose to optic structures, cGy	777 (293.5)

IQR: Interquartile range; V100: volume of tumor receiving 100% of prescription dose; V95: volume of tumor receiving 95% of prescription dose; Optic structures includes optic chiasm, left optic nerve, right optic nerve. Maximum dose was defined D1mm3, or dose received by hottest 1mm^3^ of the structure

**Fig. 1:**
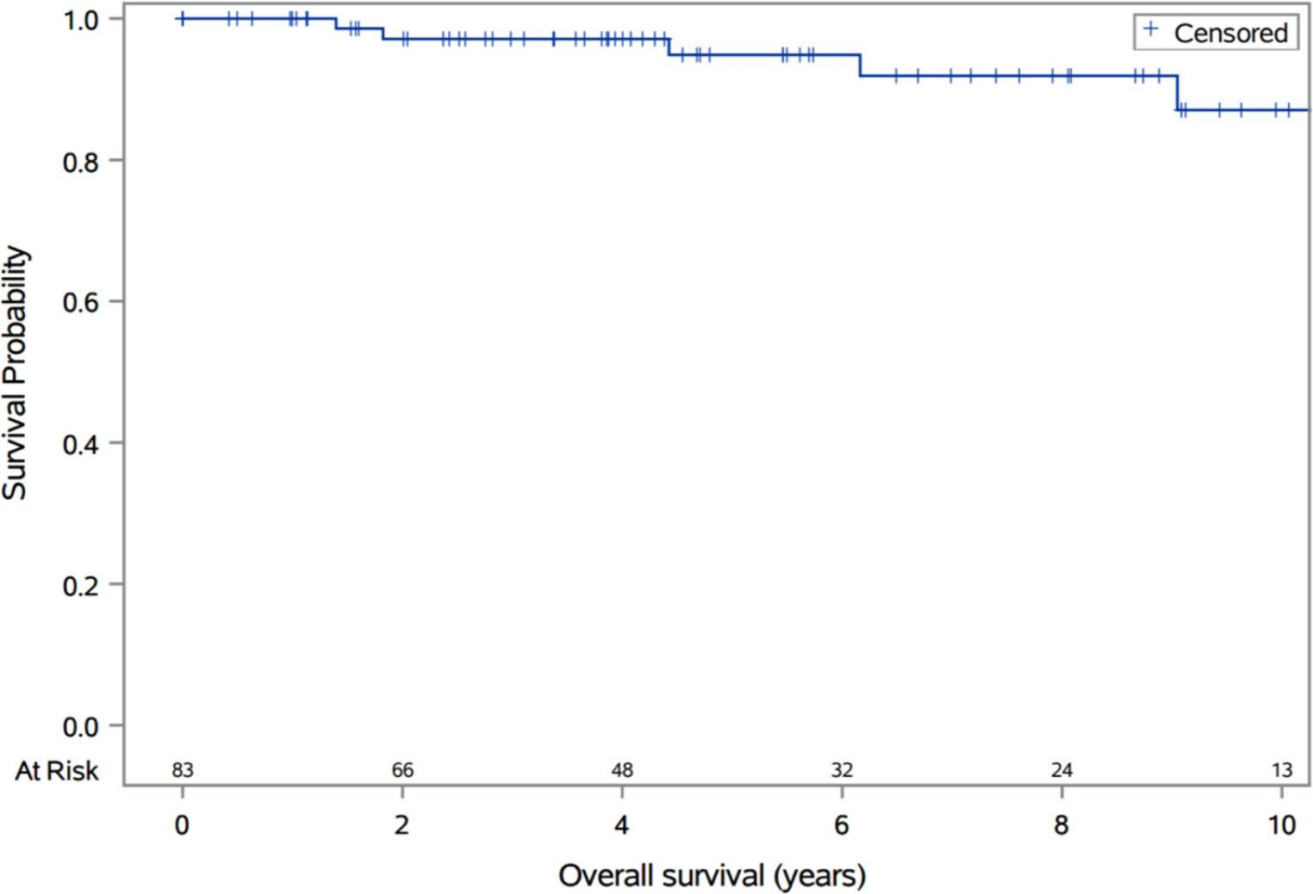
Overall survival in 83 patients following stereotactic radiosurgery. Five events (deaths) were from non-pituitary causes

With a median patient follow-up of 4.7 years, no local tumor recurrences were observed, with an estimated local control probability of 100%. Additionally, we did not observe any cases of pseudoprogression in our cohort. Two- and five-year overall survival estimates were 97% (Co) and 95% (95% CI 84–98), respectively (Fig. 1). No patient died of a cause related to their pituitary tumor. The five patients who died of non-pituitary causes succumbed to metastatic lung cancer, acute leukemia, metastatic cholangiocarcinoma, septic shock, and complications of end-stage Parkinson’s disease.

In total, 35 patients experienced hypopituitarism. Two patients presented with panhypopituitarism as their presenting complaint prior to any interventions. Twenty-one patients (60%) had hypopituitarism prior to SRS as a result of their surgical interventions. Twelve patients (34%) developed hypopituitarism after SRS. Of the patients who developed hypopituitarism after SRS, the majority developed hypothyroidism (92%), 25% developed hypogonadism, and 42% developed adrenal insufficiency. One patient developed panhypopituitarism in all 3 axes. No patient developed diabetes insipidus post-SRS.

**Fig. 2:**
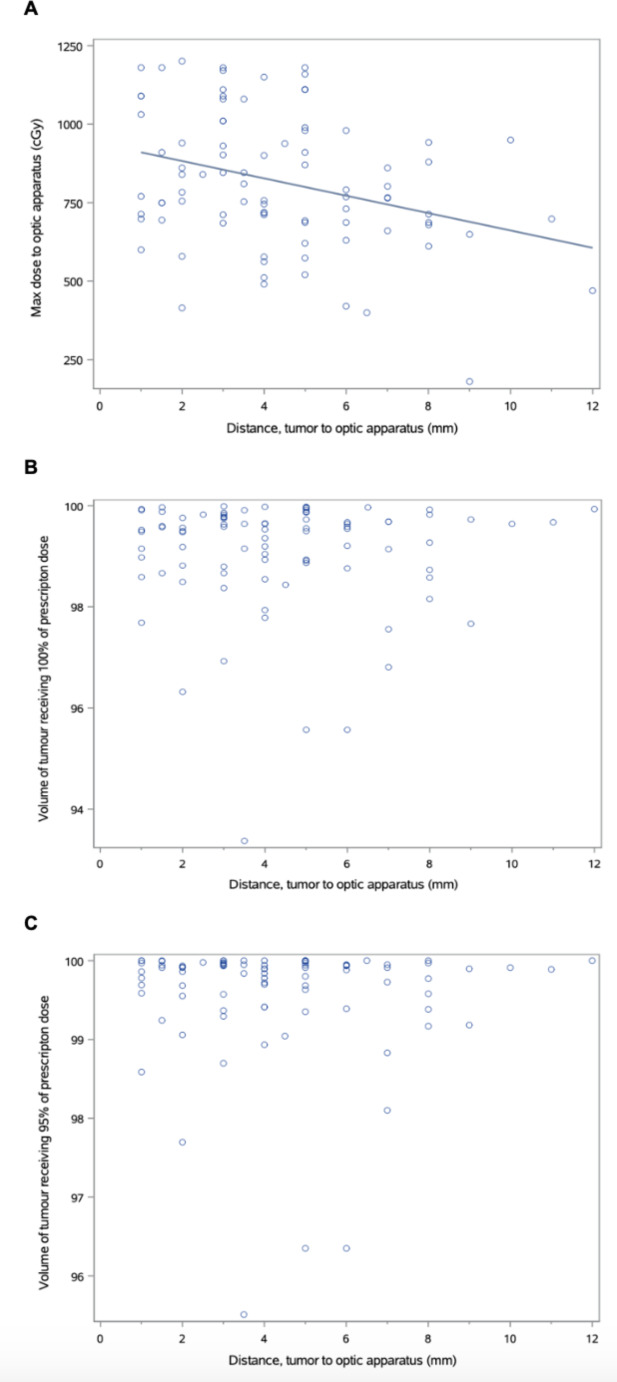
(**A**) Maximum dose (D1mm^3^) to optic apparatus decreasing with distance of tumor from apparatus. A linear regression line is shown with R^2^ = 0.10. There was no observed correlation between distance of tumor from optic apparatus and tumor coverage with (**B**) 100% of prescription dose and (**C**) 95% of prescription dose

Doses to the optic nerves and chiasm ranged from 0.4 to 12 Gy (D1mm^3^). There was a weak association between the closest distance from tumor to optic apparatus and maximum (D1mm^3^) dose to the optic structures (Fig. 2A), with increasing distance associated with a lower D1cc (R-squared value of 0.10). There was no observed association between distance from tumor to optics and coverage of the tumor by 100% of the prescription dose (Fig. 2B) or 95% of the prescription dose (Fig. 2C).

**Fig. 3:**
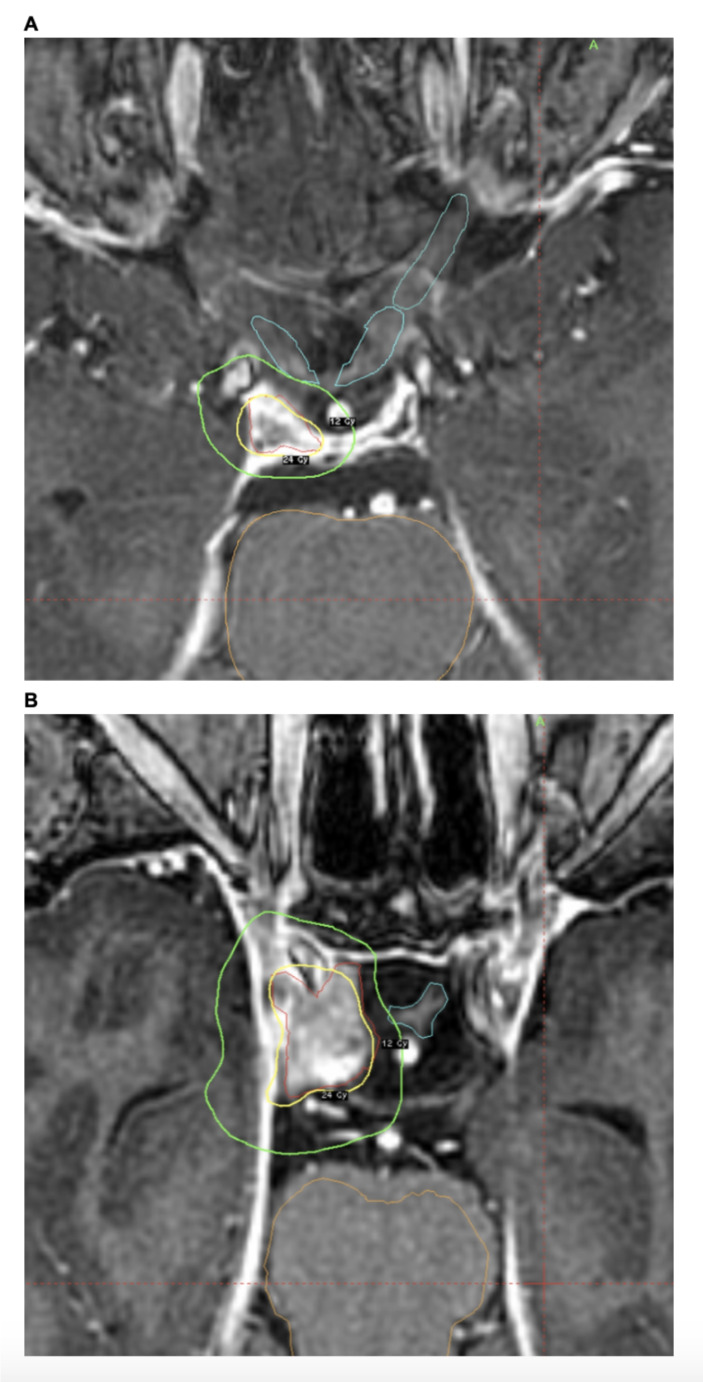
Two patients with tumor 1 mm from optic structures. (**A**) 52-year-old man with a functioning pituitary tumor treated with 2400 cGy to the 50% isodose line. The D1mm3 to chiasm was 1090 cGy. Tumor V100 was 98.59%. The patient remains alive without evidence of recurrence. (**B**) 53-year-old woman with a non-functioning pituitary tumor treated with 2400 cGy to the 56% isodose line. The D1mm3 to chiasm was 1180 cGy. Tumor V100 was 98.98%. The patient remains alive without evidence of recurrence

Eight treated lesions had a distance of 1 mm from the target to the optic apparatus. All 8 tumors had < 100% target coverage. Four of these treatments surpassed the 10 Gy threshold to optic structures. Figure 3 shows examples of treatment plans of tumors 1 mm away from optic structures. The D1mm^3^ to the chiasm was 1090 cGy (Fig. 3A) and 1180 cGy (Fig. 3B). In both cases, full tumor coverage by the prescription isodose line was achieved with careful planning. No patients experienced any optic neuropathy or visual decline post SRS based on patient-reported histories. Additionally, there were no cases of cranial neuropathy following SRS.

## Discussion

In our cohort of patients with pitNETs treated with SRS, no local recurrences were observed at a median follow-up of 4.7 years. The observed local control probability was 100%. Patients with lesions very close to the optic apparatus were included in this cohort, as 39% of patients had tumor within 3 mm of optic structures. Although some patients were treated with reduced dose coverage of the tumor, most patients were able to receive treatment with excellent dosimetric coverage, and no patients developed optic neuropathy from radiation. Moreover, selective tumor undercoverage did not lead to any local recurrences.

In a meta-analysis of 35 studies, the reported rates of local control for non-functioning pitNETs (also known as non-functioning adenomas, NFAs) were 90–100% with a median follow-up of 42 months and median dose of 15 Gy [9]. In a multi-center study of 512 nonfunctioning pitNETs, actuarial tumor control was reported to be 98%, 95%, 91%, and 85% at 3, 5, 8, and 10 years post-SRS, respectively [10]. In our study, the local control, defined as stable tumor size on post-SRS imaging, was 100% at a median follow-up of 4.7 years and median dose of 18 Gy. Our reported local control includes 26 functioning pitNETs, 6 of which achieved radiosurgical remission of excess hormone secretion (23%). The remaining functioning pitNETs achieved disease control with additional medical management following SRS.

While the accepted maximum tolerance dose to optic structures is typically 10 Gy, Leavitt et al. suggest that doses of up to 12 Gy to small portions of optic structures can be safe with a low risk (9%) of radiation-induced optic neuropathy [11]. We did not observe any cases of optic toxicities in this study with a median maximum dose to optic structures being approximately 8 Gy with some patients being treated with tumors in close (< 3 mm) proximity to optic structures. Notably, 4 patients exceeded the 10 Gy threshold and did not have neuritis. This observed finding suggests that dose de-escalation in future treatment of pitNETs could be cautiously considered.

Our findings contrast with that reported by Shen et al., who reported 41 cases of recurrent/residual non-functioning pituitary adenoma treated with gamma knife radiosurgery [3]. In their series, the authors treated pituitary tumors that were intimate with optic structures, with optic apparatus doses up to 27.6 Gy in 1 fraction. Tumor coverage was 82.5%. They reported 5 patients with visual disturbance post-treatment and 1 patient with permanent visual field defect. In addition, two patients had progressive tumor, which could be related to inadequate radiosurgery (due to proximity to optic structures) [3]. Thus, careful patient selection is required for radiosurgery; patients with tumors that are intimate with optic structures may be considered for operative resection or fractionated radiotherapy [12].

Our study does have some limitations. Firstly, our study design was limited to data from a single centre, with retrospective analysis fallible to the associated weaknesses of selection bias and applicability to the general population. This is mitigated by our large referral base, which serves the province of Ontario (2021 population 14.2 million) [13]. Secondly, patients in our institution did not undergo formal ophthalmologic assessment during follow-up, and evaluation of visual toxicities was dependent on self-reported symptoms. However, our study is strengthened by a median follow-up period nearing 5 years.

With excellent local control and no cases of optic toxicity, we suggest that dose de-escalation can be cautiously considered. We recognize the limitations of our study that the recognition of visual toxicities were dependent on patient-reported symptoms rather than objective measures such as ophthalmologic assessment. We also appreciate that our study contrasts with other reports of cases with visual disturbance post treatment. Yet, given our findings, we propose that dose de-escalation may provide patients who initially may have been limited to conventionally fractionated radiotherapy with access to SRS.

## Conclusions

In this retrospective, single-institution study of 88 tumors in 83 patients with pitNETs treated with frame-based, single-fraction, cobalt-60 stereotactic radiosurgery, we observed a local control rate of 100% with no cases of optic toxicities after a median follow-up of 4.7 years. Patients with tumor distance as low as 1 mm away from optic structures were treated successfully with radiosurgery. Our results suggest that SRS for selected patients with pitNETs as close as 1 mm from optic structures can be safely delivered with a low risk for radiation-induced optic neuropathies, and that dose de-escalation for non-functioning pitNETs may be possible given the high local control rates.

## Data Availability

Study data are available from the corresponding author upon reasonable request.
